# Comparison of joint versus purebred genomic evaluation in the French multi-breed dairy goat population

**DOI:** 10.1186/s12711-014-0067-3

**Published:** 2014-10-29

**Authors:** Céline Carillier, Hélène Larroque, Christèle Robert-Granié

**Affiliations:** INRA, UMR1388 Génétique, Physiologie et Systèmes d’Elevage, 31326 Castanet-Tolosan, France; Université de Toulouse INPT ENSAT, UMR1388 Génétique, Physiologie et Systèmes d’Elevage, 31326 Castanet-Tolosan, France; Université de Toulouse INPT ENVT, UMR1388 Génétique, Physiologie et Systèmes d’Elevage, 31076 Toulouse, France

## Abstract

**Background:**

All progeny-tested bucks from the two main French dairy goat breeds (Alpine and Saanen) were genotyped with the Illumina goat SNP50 BeadChip. The reference population consisted of 677 bucks and 148 selection candidates. With the two-step approach based on genomic best linear unbiased prediction (GBLUP), prediction accuracy of candidates did not outperform that of the parental average. We investigated a GBLUP method based on a single-step approach, with or without blending of the two breeds in the reference population.

**Methods:**

Three models were used: (1) a multi-breed model, in which Alpine and Saanen breeds were considered as a single breed; (2) a within-breed model, with separate genomic evaluation per breed; and (3) a multiple-trait model, in which a trait in the Alpine was assumed to be correlated to the same trait in the Saanen breed, using three levels of between-breed genetic correlations (ρ): ρ = 0, ρ = 0.99, or estimated ρ. Quality of genomic predictions was assessed on progeny-tested bucks, by cross-validation of the Pearson correlation coefficients for validation accuracy and the regression coefficients of daughter yield deviations (DYD) on genomic breeding values (GEBV). Model-based estimates of average accuracy were calculated on the 148 candidates.

**Results:**

The genetic correlations between Alpine and Saanen breeds were highest for udder type traits, ranging from 0.45 to 0.76. Pearson correlations with the single-step approach were higher than previously reported with a two-step approach. Correlations between GEBV and DYD were similar for the three models (within-breed, multi-breed and multiple traits). Regression coefficients of DYD on GEBV were greater with the within-breed model and multiple-trait model with ρ = 0.99 than with the other models. The single-step approach improved prediction accuracy of candidates from 22 to 37% for both breeds compared to the two-step method.

**Conclusions:**

Using a single-step approach with GBLUP, prediction accuracy of candidates was greater than that based on parent average of official evaluations and accuracies obtained with a two-step approach. Except for regression coefficients of DYD on GEBV, there were no significant differences between the three models.

## Background

With the recent availability of the Illumina goat SNP50 BeadChip [1], it has become possible to study the implementation of genomic selection in French dairy goat. In this species, as in dairy cattle, genomic selection has the ability to shorten the generation interval for the sire-son pathway (5.5 years with progeny-testing [2]) by selecting males shortly after birth. The utility of genomic selection is dictated by the accuracy of the genomic breeding values (GEBV) of the selection candidates, which has to be greater than the parent average accuracy for genomic ($\sqrt{\frac{1}{4}\ \mathrm{reliability}\ \mathrm{sire}+\frac{1}{4}\ \mathrm{reliability}\ \mathrm{dam}}$) selection to be effective.

However, although the French population of genotyped goats is the largest worldwide, it only counts around 900 males of the Alpine and Saanen breeds. Analysis of the genetic structure of this population [3], based on estimates of linkage disequilibrium and inbreeding and kinship coefficients, showed a high level of genetic diversity. The number of effective founders was estimated to be equal to 191 and 124 for the Alpine and Saanen breeds, respectively, based on females born between 2010 and 2011 [2]. Considering the relatively small sample size and the genetic structure of this population, the prediction accuracy of GEBV is not expected to be as high in these populations as in dairy cattle [4].

Within the French dairy goat breeding scheme, bucks used for breeding have more than 2000 daughters at the end of their productive life. Females used as buck dams have at least three lactations. This yields accurate parent estimated breeding values (EBV), and consequently fairly high parent average accuracies of young selection candidates (0.63 on average for milk yield) [3].

A first study on genomic prediction in dairy goats [3] used a two-step genomic approach. Step one consisted of deriving average daughter performance corrected for fixed and non-genetic random effects and for genetic effects of the dams (daughter yield deviations, DYD). Step two involved a genomic evaluation based on these DYD. These steps are especially essential for dairy traits on which males are selected and genotyped but for which they are not phenotyped [5]. This approach resulted in GEBV with accuracies as high as those of parent average EBV for young selection candidates that were not yet progeny-tested [3].

GEBV accuracies of candidates depend on the genetic characteristics of the reference population i.e. number of individuals, effective population size, linkage disequilibrium, inbreeding level, and relationship of candidates to the reference population and heritability of the phenotype [6-8]. The structure of the reference population could not be optimized by either genotyping other males because all progeny-tested males were genotyped or by choosing in the reference population the highly related males because of its size. In this context, GEBV accuracy could only be improved by using the most suitable model for genomic evaluation. Several studies [9-11] have shown that the single-step approach proposed by Legarra et al. [12] provides greater accuracy than the two-step approach used in [3]. The single-step approach allows all recorded phenotypes to be used, without pre-adjustment for fixed effects, and to evaluate all animals, regardless of whether they have been genotyped or phenotyped [5]. Another approach, the pseudo-single-step approach [11] can also deal with non-genotyped individuals by adding records of non-genotyped males in a two-step procedure. The pseudo-single-step approach is a two-step approach (i.e. based on DYD). However, it is an intermediate approach between the two-step and single-step approaches, because it includes information on all males with DYD, but ignores maternal information unlike the single-step approach [11]. Thus, it was expected that validation correlations using the pseudo-single-step approach would be intermediate to those obtained with the single-step and two-step approaches.

Our goat population was composed of two breeds that were evaluated together in the previous study because of their small population sizes [3]. These breeds are managed together in 10% of the French dairy goat herds. The white coat Saanen breed is a selected variety of the Alpine breed that originated several centuries ago [13]. Currently, selection in French dairy goats is done within-breed for the Alpine and Saanen breeds based on within-breed genetic evaluations, except for milk production traits, for which a multi-breed model is used. Alpine and Saanen breeds have similar heritability and repeatability parameters for milk production and type traits but differ in genetic and residual variances [14], persistence of linkage disequilibrium [3] and allele frequencies. These differences raise doubts on the benefits of multi-breed genomic evaluation for this population. Few studies dealing with multi-breed evaluation in other species have compared their results to within-breed models [15-17]. When several breeds are pooled in a single reference population, accuracies depend on the genetic characteristics and similarities between breeds. Recent research has not led to a consensus on the advantage of using multi-breed genomic evaluation. Very few studies have explored the use of relationships between breeds in multi-breed genomic evaluation [18,19] using, for instance, multiple-trait models.

Here, we tested a single-step approach using three models. The first model was a multi-breed model in which Alpine and Saanen populations were pooled together and considered as a single population (with only one set of genetic parameters). The second model was a multiple-trait model as described in Karoui et al. [18], where a trait recorded in the Alpine breed was considered to be different from, but correlated with, the same trait recorded in the Saanen breed. In this case, genetic parameters were specific to each breed. The third model was a within-breed model, where one model was used for the Saanen population and another for the Alpine population, with different genetic parameters for each breed.

## Methods

### Data

The SNP genotypes obtained using DNA samples extracted from blood were performed according to the French National Guidelines for the care and use of animals for research purposes. Animal genotypes used in this study were the same as in Carillier et al. [3]. After a quality check (MAF > 1%, call rate > 98%) that was done separately for the two breeds, 46 959 SNPs (out of 53 347 of the Illumina SNP50 BeadChip [1]) were validated. Missing SNP genotypes (0.1%) were not imputed but the GBLUP method used took missing data into account when estimating GEBV. From the 825 genotyped bucks (355 Saanen and 470 Alpine individuals) born between 1993 and 2011, 148 (86 Alpine and 62 Saanen individuals born in 2010 and 2011) were not yet progeny-tested and could not be used for training.

Five milk production traits that were derived from a total lactation were analyzed, i.e. milk yield (kg), fat and protein yields (kg), fat and protein contents (g/kg), along with somatic cell score (SCS: log-transformed somatic cell counts), and five udder type traits that were scored on a linear scale of 1 to 9, i.e. udder floor position, udder shape, rear udder attachment, fore udder and teat angle. Udder type traits were recorded once for each female. Repeatability was not modeled for the udder type traits. Data originated from the official genetic evaluation of January 2013, using only records on purebred Alpine and Saanen goats. For milk production traits, 4 178 315 Alpine records (30.2% first lactations, 24.2% second lactations and 45.6% third or more lactations) and 3 173 516 Saanen records (31.1% first lactations, 24.5% second lactations and 44.4% third or more lactations) of females born between 1950 and 2012 were used. Recently, the number of records for SCS and type traits has been smaller than that for milk production traits. Weights for SCS and milk production records were as defined in the official genetic evaluation [20,21] according to lactation number (from 1 to 10) and length of lactation (up to 180 days or not).

The pedigree used in this study consisted of 2 981 809 animals (40% Saanen, 53% Alpine, 4% crossbred of Alpine and Saanen and 3% other breeds) born between 1950 and 2012 and considered up to 29 generations for males. It was completed by 43 unknown parent groups defined according to breed and birth year: one group every two years, with sires and dams pooled together.

### Genetic models used for analysis

Animal GEBV were estimated using genomic best linear unbiased prediction (BLUP) with the blup90iod program [22] , using models as described in the following.

#### Multi-breed model

The first model used for multi-breed genomic prediction was:

$$ \mathbf{y}=\mathbf{X}\boldsymbol{\upbeta } +\mathbf{Z}\mathbf{u}+\mathbf{W}\mathbf{p}+\mathbf{e}, $$

where **y** is the vector of all female records (ν) from the two breeds weighted by their lactation weights as defined above, and **X** is the incidence matrix relating fixed effects (**β**) to individuals. The following fixed effects were considered for milk production traits and SCS: herd (within year and parity), age and month at delivery (within year and region), length of dry period (within year and region) and breed. For type traits, fixed effects were: herd (within year), age at scoring, lactation stage and breed. **W** is the incidence matrix relating permanent environmental effects (**p**), which were normally distributed $N\left(\mathbf{0},{\upsigma}_p^2{\mathbf{I}}_t\right)$, to individuals, **Z** is a design matrix allocating observations to breeding values (**u**), and **e** is a vector of random normal errors, normally distributed $N\left(\mathbf{0},{\upsigma}_e^2{\mathbf{I}}_{\mathrm{v}}\right)$. Genomic breeding values **u** were assumed normally distributed with $Var\left(\mathbf{u}\right)=\mathbf{H}{\upsigma}_u^2$, where **H** is the multi-breed genetic relationship matrix combining SNP marker information and pedigree data, implemented as in Legarra et al. [12]:

$$ \mathbf{H}=\left(\begin{array}{cc}\hfill {\mathbf{A}}_{11}+{\mathbf{A}}_{12}{\mathbf{A}}_{22}^{-1}\left(\mathbf{G}-{\mathbf{A}}_{22}\right){\mathbf{A}}_{22}^{-1}{\mathbf{A}}_{21}\hfill & \hfill {\mathbf{A}}_{12}{\mathbf{A}}_{22}^{-1}\mathbf{G}\hfill \\ {}\hfill \mathbf{G}{\mathbf{A}}_{22}^{-1}{\mathbf{A}}_{21}\hfill & \hfill \mathbf{G}\hfill \end{array}\right), $$

where **A**_**11**_ is a sub-matrix of the pedigree-based relationship matrix (**A**) for ungenotyped animals, **A**_**22**_ is a sub-matrix for genotyped animals, and **A**_**12**_ (or **A**_**21**_) is a sub-matrix that describes the pedigree-based relationship between ungenotyped and genotyped animals. The genomic relationship matrix (**G**) was scaled to be comparable with the **A** matrix using the correction defined by Gao et al. [10]. Matrix **G** was derived as in [23].

$$ \mathbf{G}=\frac{\mathbf{M}{\mathbf{M}}^{\mathbf{\prime}}}{2{\displaystyle \sum_{\mathrm{j}=1}^{\mathrm{p}}{\mathrm{q}}_{\mathrm{j}}\left(1-{\mathrm{q}}_{\mathrm{j}}\right)}}, $$

where *p* is the number of SNPs, q_j_ the allele frequency of the whole population (Alpine and Saanen) for SNP j and **M** is a centered matrix of SNP genotypes.

#### Within-breed model

The within-breed model was similar to the above multi-breed model except that Alpine and Saanen were evaluated separately. The relationship matrices (**H**_s_ for Saanen breed and **H**_a_ for Alpine breed) were defined as in the previous model except that they were derived from the allele frequencies (q_j_) observed in each breed. The same pedigree as defined above in the paragraph on data was used to derive the pedigree relationship matrix.

#### Multiple-trait model

The third model used in this study was the same as that used in [18]:

$$ {\mathbf{y}}_b={\mathbf{X}}_b{\boldsymbol{\upbeta}}_b+{\mathbf{Z}}_b{\mathbf{u}}_b+{\mathbf{W}}_b{\mathbf{p}}_b+{\boldsymbol{\upvarepsilon}}_b, $$

where $$ {\mathbf{y}}_b=\left(\begin{array}{c}\hfill {\mathbf{y}}_a\hfill \\ {}\hfill {\mathbf{y}}_s\hfill \end{array}\right) $$ and $$ {\mathbf{u}}_b=\left(\begin{array}{c}\hfill {\mathbf{u}}_{b_a}\hfill \\ {}\hfill {\mathbf{u}}_{b_s}\hfill \end{array}\right) $$ is the vector of true breeding values normally distributed with $$ Var\left({\mathbf{u}}_b\right)=\left(\begin{array}{cc}\hfill {\upsigma}_{u_a}^2\hfill & \hfill {\upsigma}_{u_{s,a}}\hfill \\ {}\hfill {\upsigma}_{u_{s,a}}\hfill & \hfill {\upsigma}_{u_s}^2\hfill \end{array}\right)\otimes \mathbf{H} $$. The fixed effects considered in this model $$ {\boldsymbol{\upbeta}}_b=\left(\begin{array}{c}\hfill {\boldsymbol{\upbeta}}_{b_a}\hfill \\ {}\hfill {\boldsymbol{\upbeta}}_{b_s}\hfill \end{array}\right) $$ were the same as in the within-breed model. The vector of permanent environmental effects, $$ {\mathbf{p}}_b=\left(\begin{array}{c}\hfill {\mathbf{p}}_{b_a}\hfill \\ {}\hfill {\mathbf{p}}_{b_s}\hfill \end{array}\right) $$, was normally distributed with $$ Var\left(\mathbf{p}b\right)=\left(\begin{array}{cc}\hfill {\upsigma}_{p_a}^2{\mathbf{I}}_{t_1}\hfill & \hfill \mathbf{0}\hfill \\ {}\hfill \mathbf{0}\hfill & \hfill {\upsigma}_{p_s}^2{\mathbf{I}}_{t_2}\hfill \end{array}\right) $$, where ${\upsigma}_{p_a}^2$ and ${\upsigma}_{p_s}^2$ are the values used in official evaluations (Table 1). The genomic relationship matrix was built as in the multi-breed model and estimated with allele frequencies derived across breed. In this model, $$ {\boldsymbol{\upvarepsilon}}_b=\left(\begin{array}{c}\hfill {\boldsymbol{\upvarepsilon}}_{b_a}\hfill \\ {}\hfill {\boldsymbol{\upvarepsilon}}_{b_s}\hfill \end{array}\right) $$ is the vector of random normal errors, defined as: $$ Var\left({\boldsymbol{\upvarepsilon}}_b\right)=\left(\begin{array}{cc}\hfill {\upsigma}_{\upvarepsilon_a}^2{\mathbf{I}}_{n_1}\hfill & \hfill \mathbf{0}\hfill \\ {}\hfill \mathbf{0}\hfill & \hfill {\upsigma}_{\upvarepsilon_s}^2{\mathbf{I}}_{{\mathrm{n}}_2}\hfill \end{array}\right). $$

**Table 1:** **Genetic parameters used in the multi-breed model**

**Trait**	**Genetic variance**	**Heritability**	**Repeatability**
Milk yield	11665.48	0.30	0.50
Fat yield	14.26	0.30	0.50
Protein yield	9.04	0.30	0.50
Fat content	8.73	0.50	0.70
Protein content	2.32	0.50	0.70
Somatic cell score	0.30	0.20	0.47
Udder floor position	0.30	0.31	-
Udder shape	0.59	0.34	-
Rear udder attachment	0.42	0.27	-
Fore udder	0.34	0.29	-
Teat angle	0.20	0.29	-

Repeatability is not modeled for udder type traits.

Three levels of genetic covariance between the traits for the Alpine and Saanen breeds (${\upsigma}_{u_{s,a}}$) were used in this study: (1) the covariance estimated from the data (see the section on “Genetic parameter estimation” below), (2) the covariance such that the genetic correlation (ρ) was equal to 0, which leads to a model that is similar to the within-breed model, and (3) the covariance such that the genetic correlation was equal to 0.99, which results in a model that is close to the multi-breed model.

### Estimation of genetic parameters

The genetic parameters used in the multi-breed model were those used in official genetic evaluations (Table 1). For the multiple-trait and within-breed models, genetic parameters were estimated from the data (Table 2), except for repeatabilities, which were those in Table 1 and considered similar for both breeds.

**Table 2:** **Estimates of genetic parameters of type and production traits in Saanen and Alpine goat population using the multiple-trait model**

**Trait**	**Alpine goats**		**Saanen goats**		**Estimated genetic correlation between Alpine and Saanen**
	**Genetic variance**	**Heritability**	**Genetic variance**	**Heritability**	
Milk yield	9, 962	0.31	7, 278	0.26	0.45
Fat yield	14.1	0.28	11.2	0.25	0.48
Protein yield	8.6	0.31	6.1	0.25	0.54
Fat content	7.3	0.48	8.0	0.51	0.46
Protein content	2.3	0.60	2.9	0.56	0.54
Somatic cell score	0.3	0.20	0.3	0.16	0.47
Udder floor position	0.6	0.51	0.6	0.57	0.76
Udder shape	0.7	0.40	0.9	0.47	0.59
Rear udder attachment	1.2	0.47	1.1	0.52	0.73
Fore udder	0.6	0.44	0.4	0.42	0.59
Teat angle	0.3	0.42	0.4	0.45	0.66

Genetic and residual variances ${\upsigma}_{u_s}^2$, ${\upsigma}_{u_a}^2$, ${\upsigma}_{e_s}^2$ and ${\upsigma}_{e_a}^2$ and the genetic covariance were estimated by restricted maximum likelihood (REML) with remlf90 software [22], using a multiple-trait model (see above) and the multi-breed **H** matrix described earlier. Standard deviations of heritabilities and genetic correlations were estimated using the approximation of Klei [24], using airemlf90 software [22]. Because of computational issues, we chose to estimate these parameters on a subset of first lactation records from the data, without considering repeatability. For milk production traits, these parameters were estimated using 113 980 first lactation records (49 201 for the Saanen and 64 779 records for the Alpine breed) from the population of all females born between 2008 and 2012. Since the data included fewer records for SCS and udder type traits, all first lactation records on all females born between 2002 and 2012 were used for these traits (i.e. 130 230 records for SCS and 202 102 for udder type traits). These analyses included 1985 females genotyped with the Illumina SNP50 BeadChip [1], using the same quality control as for males. Genotyped females were groups of 100 half-sibs from 20 different sires [3] involved in a design for quantitative trait locus (QTL) detection [25].

### Cross-validation analyses

Cross-validation consisted in splitting the population of 677 progeny-tested and genotyped bucks into two sets: the training set (425 males born between 1993 and 2005: 232 Alpine and 193 Saanen individuals) and the validation set (252 males born between 2006 and 2009: 100 Saanen and 152 Alpine individuals). Phenotypes used for the training set were records of females born before 2008, i.e. the first year of lactation for daughters of bucks born in 2005. Prediction quality was evaluated based on Pearson correlations between GEBV and DYD [26] for the validation males, and regression coefficients of DYD on GEBV. The DYD were obtained from official genetic evaluations (January 2013). These validation correlations serve as indicators of predictive ability and the regression coefficients (slopes) serve as indicators of the dispersion of GEBV; a slope above 1 indicates under-dispersion of GEBV and a slope below 1 indicates over-dispersion.

Prediction error variances (PEV) of GEBV were estimated as in Misztal et al. [27] by estimating the inverse of the coefficients matrix of the mixed-model equations [28] using FORTRAN program accf90. Average model accuracies were derived from PEV as in Carillier et al. [3] for the 148 young males that were not yet progeny-tested and born between 2010 and 2011. Prediction quality of validation males, and GEBV accuracy of young animals were analyzed both in the whole population (Alpine + Saanen animals) and also for each breed separately.

## Results and discussion

### Genetic parameters

Table 2 reports the estimates of heritability (h^2^), genetic variances and genetic correlations between Alpine and Saanen breeds for all traits studied. Standard errors of heritability ranged from 0.008 for udder type traits for the Alpine breed to 0.020 for milk production traits for the Saanen breed (results not shown). The highest heritabilities were found for protein content (around 0.6), udder depth (around 0.55), rear udder attachment (around 0.5) and fat content (0.5). SCS was the less heritable trait, with estimates of 0.2 and 0.16 in the Alpine and Saanen breeds, respectively.

Heritability estimates obtained for milk production traits were close to those reported by Belichon et al. [14] but they tended to be smaller, especially for fat yield in Alpine goats (0.28 *vs* 0.37), protein yield in Saanen goats (0.25 *vs* 0.34), and fat content in both breeds (0.48 *vs* 0.58 and 0.51 *vs* 0.60 in Alpine and Saanen goats, respectively). Our heritability estimates for udder type traits were much higher than those reported by Clément et al. [20]: 0.51 and 0.57 *vs* 0.34 and 0.37 for udder floor position in the Alpine and Saanen breeds, respectively. These differences could be explained by the more recent data that were used here, with females born between 2008 and 2012, compared to the females born between 1998 and 1997 [14] and 2000 and 2002 [20] in other studies. Genetic parameter estimates for SCS were fairly similar to those reported by Rupp et al. [29].

Heritability estimates were similar for Saanen and Alpine goats except for udder shape (0.47 *vs* 0.40), udder floor position (0.57 *vs* 0.51) and protein yield (0.25 *vs* 0.31) (Table 2). The largest between-breed differences in heritability were previously reported for udder shape (0.40 *vs* 0.28 in Alpine and Saanen goats, respectively) and protein content (0.58 *vs* 0.50) [14,20]. However, genetic variances in our study tended to differ between Alpine and Saanen populations (14.1 *vs* 11.2 for instance for fat yield). Thus, the similar heritability estimates in the two breeds were explained by a similar ratio between genetic and residual variances, rather than similar variances.

Estimates of the genetic correlation between traits in the Alpine and Saanen breeds ranged from 0.45 for milk yield to 0.76 for udder floor position (Table 2). Standard errors of estimates of the genetic correlation ranged from 0.1 for udder type traits and protein content to 0.3 for SCS (results not shown). Estimates were close to those estimated between Holstein and Normande and between Montbéliarde and Normande dairy cattle breeds [18], i.e. from 0.38 to 0.46 for milk yield and from 0.35 to 0.56 for fat content, but lower than the correlations between Holstein and Montbeliarde breeds (0.79 for milk yield and 0.66 for fat content). These results suggest that French Holstein and Montbeliarde dairy cattle breeds are genetically closer than the Alpine and Saanen goat breeds, perhaps due to the introgression of Red Holstein genes in the Montbéliarde breed in the 1980’s. The highest correlations and the lowest standard errors of these correlations were obtained for udder floor position (0.76) and rear udder attachment (0.73). These high genetic correlations for udder type traits suggest that marker effects in the Alpine breed were closer to marker effects in the Saanen breed for these particular traits.

### Analysis of the multi-breed population

#### Validation correlations

To make results from the models comparable, GEBV from the within-breed model were centered on the overall average across the two breeds. For the within-breed model, validation correlations and slopes (Tables 3 and 4) were estimated using the centered GEBV of all validation males (Alpine + Saanen).

**Table 3:** **Pearson correlations between DYD and GEBV from three models for the 252 validation males regardless of breed**

**Trait**	**Multi-breed**	**Multiple-trait**			**Within-breed**
		**ρ = 0.99**	**ρ estimated**	**ρ = 0**	
Milk yield	0.43	0.43	0.42	0.43	0.43
Fat yield	0.44	0.45	0.46	0.46	0.46
Protein yield	0.33	0.36	0.35	0.35	0.36
Fat content	0.61	0.59	0.60	0.60	0.63
Protein content	0.70	0.68	0.70	0.70	0.69
Somatic cell score	0.47	0.47	0.47	0.47	0.47
Udder floor position	0.59	0.59	0.59	0.59	0.59
Udder shape	0.55	0.56	0.56	0.56	0.56
Rear udder attachment	0.64	0.66	0.66	0.67	0.66
Fore udder	0.50	0.49	0.50	0.51	0.51
Teat angle	0.61	0.60	0.58	0.57	0.59

ρ is the genetic correlation between Alpine and Saanen goats used in the multiple-trait model.

**Table 4:** **Regression coefficients of DYD on GEBV from three models for the 252 validation males regardless of breed**

**Trait**	**Multi-breed**		**Multiple-trait**						**Within-breed**	
			**ρ = 0.99**		**ρ estimated**		**ρ = 0**			
	**reg**	**SE**	**reg**	**SE**	**reg**	**SE**	**reg**	**SE**	**reg**	**SE**
Milk yield	0.58	0.12	0.76	0.10	0.48	0.10	0.49	0.10	0.77	0.10
Fat yield	0.62	0.08	0.67	0.08	0.62	0.08	0.61	0.08	0.69	0.08
Protein yield	0.43	0.09	0.53	0.09	0.44	0.09	0.44	0.09	0.54	0.09
Fat content	0.86	0.07	0.97	0.08	0.76	0.08	0.76	0.08	0.79	0.08
Protein content	0.95	0.07	0.92	0.07	0.94	0.06	0.94	0.07	1.17	0.08
Somatic cell score	0.67	0.08	0.68	0.08	0.64	0.08	0.64	0.08	0.71	0.08
Udder floor position	0.76	0.08	0.86	0.07	0.80	0.08	0.80	0.08	0.90	0.08
Udder shape	0.66	0.09	0.96	0.09	0.65	0.09	0.65	0.09	0.96	0.09
Rear udder attachment	0.70	0.09	1.46	0.11	0.60	0.10	0.59	0.10	1.51	0.11
Fore udder	0.60	0.09	0.89	0.10	0.55	0.10	0.56	0.10	0.94	0.10
Teat angle	0.74	0.08	1.10	0.09	0.58	0.09	0.56	0.11	1.19	0.10

ρ is the genetic correlation between Alpine and Saanen goats used in the multiple-trait model.

Table 3 reports the Pearson correlations between GEBV and DYD for the 252 validation males for all models. The correlations ranged from 0.33 for protein yield using the multi-breed model to 0.70 for protein content using the multi-breed or the multiple-trait model with ρ estimated or equal to 0. The highest correlations were obtained for traits with the highest heritabilities, i.e. for fat content (0.61), protein content (0.70), and rear udder attachment (0.64), as previously also reported by Carillier et al. [3].

Using the within-breed model, validation correlations were slightly greater than with the multi-breed model, except for protein content (−1%) and teat angle (−3%). The largest increase was for protein yield (+9%). Other studies using within-breed models [17,19,30] did not evaluate regression slopes and correlations of GEBV across breeds, which makes it difficult to compare results with our findings. The studies that compared multi-breed to within-breed genomic evaluations reported increases in validation correlations from 2% for milking ability in Finnish cattle to 50% for maternal calving in Swedish cattle [17] and from 52% for milk yield to 80% for protein yield in Chinese bulls [30]. However, grouping several breeds (Holstein, Jersey and Brown Swiss) in the same training set could reduce validation correlations by 2% to 3% [19].

Validation correlations obtained with the multiple-trait models were close to those obtained with the within-breed and multi-breed models, with differences ranging from 1% for udder shape to 6% for fat content. In dairy cattle, Olson et al. [19] found that the multiple-trait model outperformed (+9%) the multi-breed model in a two-step approach on de-regressed EBV. Using different values for the genetic correlation between Alpine and Saanen breeds (ρ = 0.99, ρ estimated and ρ = 0) did not have a major impact on correlations between DYD and GEBV (Table 3). Differences ranged from 0% for SCS, udder floor position and udder shape, to 3.4% for teat angle. These results are consistent with those of Karoui et al. [18] who observed no impact of the genetic correlation on validation correlations and regression coefficients.

Comparison of the results obtained with the multi-breed model and with two-step approach [3] shows that validation correlations using the single-step approach increased for all traits, by 10% for milk yield up to 74% for teat angle, expect for protein yield (−8%). Increases were greater for udder type traits (mean 59%) than for milk production traits (mean 14%). This is consistent with results found in a Lacaune dairy sheep population [11,31], for which validation correlations increased from 11% for milk yield to 53% for udder depth. Previous studies using the pseudo-single-step approach led to intermediate results with increases in validation correlations from 0.1% to 10% in Nordic Holstein cattle [10] and from 2% to 34% in Lacaune dairy sheep [11] compared to the two-step approach.

#### Regression coefficients of DYD on GEBV

Estimates of the regression coefficients of DYD on GEBV for validation males (Table 4) ranged from 0.43 for protein yield using the multi-breed model to 1.51 for rear udder attachment using the within-breed model. Standard errors of these estimates ranged from 0.06 for protein content with the multiple-trait model using the estimated ρ to 0.12 for milk yield with the multi-breed model. Based on these standard errors, no significant differences in slopes were observed between the models. The lowest regression coefficients of DYD on GEBV were found for protein yield (0.43 with the multi-breed model), fore udder (0.55 with the multiple-trait model using the estimated ρ) and fat yield (0.61 with the multiple-trait model using ρ = 0). Slopes that were the furthest from 1 were obtained for traits with the lowest validation correlations between DYD and GEBV. It is difficult to interpret these slopes when the estimated validation correlations are not sufficient. However, slopes of DYD on GEBV less than 1, which indicates over-dispersion of GEBV, were observed for almost all traits except for protein content with the within-breed model, and for rear udder attachment and teat angle with the multiple-trait model using ρ = 0.99, as well as with the within-breed model for these three traits.

Regression coefficients were slightly closer to 1 with the within-breed model than with the multi-breed model, i.e. by +6% for SCS to +57% for fore udder, except for fat (−8%) and protein contents and for rear udder attachment (Table 4). These results indicate less dispersion of GEBV with the within-breed model than with the multiple-trait model for almost all traits. Using the multiple-trait model, slopes were slightly greater when ρ was estimated or equal to 0 (by 1% for fat yield with ρ = 0 to 48% for fore udder with ρ estimated) compared to the model with ρ = 1, as in the multi-breed model, except for some traits. Regression coefficients that were obtained with a genetic correlation estimated or equal to 0 were similar (equal or up to 2% different; Table 4). The slopes obtained with ρ = 0.99 were consistent with those obtained with the two-step approach [3], at 0.76 *vs* 0.79 for example for milk yield. Using a genetic correlation of 0.99 greatly reduced the dispersion of the GEBV compared to other correlation levels, i.e. by 6% for udder shape to up to 61% for teat angle, but not for protein content or rear udder attachment. In the study by Karoui et al. [18], slopes that were estimated using a between-breed genetic correlation of 0.95 were similar to those obtained with an estimated genetic correlation ranging from 0.38 to 0.79 for milk yield depending on the breeds considered.

Regression coefficients obtained with the multi-breed single-step model were lower than those estimated with the two-step approach [3], by 9% for udder floor position and by up to 43% for protein yield, except for protein content. The differences obtained in this study were consistent with those reported in the literature: 17% for final score in US Holstein bulls [5], and from 12% for milk yield to 14% for SCS in Lacaune dairy sheep [11,31]. These regression coefficients were not as good as expected, although allele frequency differences between the genotyped and base-population animals [5] were taken into account in the genomic relationship matrix using the approach of Christensen [32]. The correction of the genomic relationship matrix for differences between base-population and genotyped animals proposed by Vitezica et al. [33] gave similar results for slopes of DYD on GEBV (results not shown). Gao et al. [10,34] showed that in a Nordic Holstein dairy cattle population, the corrections done to the genomic relationship matrix as proposed in [33] did not significantly reduce the over-dispersion of GEBV (from 0% to 3%) and even increased it in some cases (from 1% to 2%).

#### Model-based accuracies

**Figure 1:**
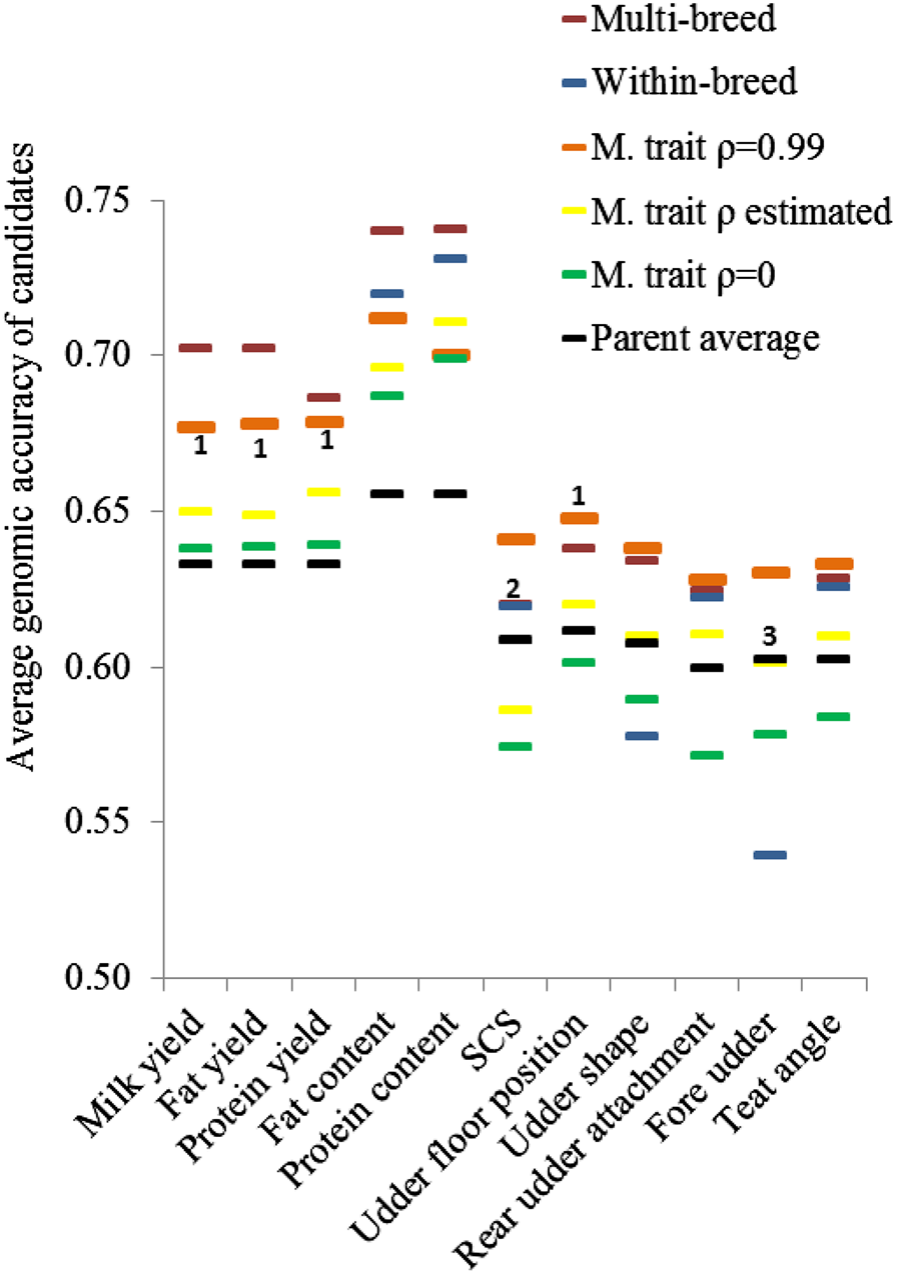
**Average model accuracy for the 148 candidate bucks regardless of breed.** 1 means similar results were obtained for the within-breed model and the multiple-trait model using ρ = 0.99; 2 means similar results were obtained for the within-breed model and the multi-breed model; 3 means similar results were obtained for the multi-breed model and the multiple-trait model using ρ = 0.99. M. trait means multiple-trait model.

Figure 1 shows the average model accuracies estimated on the 148 candidates using predictions based on the 677 males of the reference population. These accuracies ranged from 0.54 for fore udder using the within-breed model to 0.74 for fat and protein contents using the multi-breed model. The highest accuracies (on average 0.5 and 0.56 for fat and protein content, respectively) were obtained for traits with the highest heritabilities.

With the within-breed model, model accuracies were similar to those obtained with the multiple-trait model at ρ equal to 0.99 for milk, fat and protein yields and for udder floor position (Figure 1). However, they were lower than with the multi-breed model, except for udder depth (+1.5%) and SCS (the same result was obtained with the three models). Reductions in accuracy when using the within-breed model compared to the multi-breed model ranged from −1% for protein content to −14% for fore udder. These results could be explained by the small size of the reference population of each breed. With the multiple-trait model, average model accuracy was higher when ρ was set equal to 0.99 than with the estimated ρ, which in turn was higher than with ρ set equal to 0. Accuracies with the multiple-trait model were lower than with the multi-breed model by −1% for protein yield to −5% for protein content, but slightly higher (by +1% to +3%) for teat angle, udder shape and rear udder attachment.

The best model-based accuracies were obtained using the multi-breed model for milk production traits and using the multiple-trait model with ρ equal to 0.99 for SCS and udder type traits (Figure 1). Compared to the two-step approaches [3], the single-step approach increased model accuracy from 28% for udder type traits and SCS to 37% for milk, fat and protein yields. These model accuracies were greater than parent average accuracies for almost all traits, by 1% for rear udder attachment and teat angle with the multiple-trait model and the estimated ρ, to 14% for fat and protein contents using the multi-breed model. However, model-based accuracies did not exceed parent average accuracy for: (1) SCS and udder type traits with the multiple-trait model and ρ set to 0, (2) udder shape and fore udder with the within-breed model, and (3) SCS with the multiple-trait model and the estimated ρ.

### Analysis by breed

In Tables 5 and 6, GEBV from the multi-breed model were deviated from the mean GEBV of each breed to compare results from the multi-breed and the within-breed models. Table 5 shows Pearson correlations between GEBV and DYD for the 152 Alpine and 100 Saanen validation males using the within-breed and the multi-breed models. Correlations obtained with the multiple-trait models were similar to those with the multi-breed model (results not shown), which is consistent with Makgahlela et al. [34]. The validation correlations were similar for the multi-breed and the within-breed models, except for fat yield in Saanen, protein yield in Alpine, and fat content in both breeds. In dairy cattle, combining several breeds in the training set did not improve validation correlations [19,35] except for the Brown Swiss breed, which had the smallest population size [19]. The higher prediction ability obtained with the within-breed model compared to the multi-breed model (Table 5) for fat content (0.55 vs 0.47 in Alpine and 0.65 *vs* 0.53 in Saanen populations) could be explained by the presence of one of the mutations in the *DGAT1* gene in the Saanen breed but not in Alpine goats (C Maroteau, UNCEIA, Toulouse, personal communication).

**Table 5:** **Breed-specific Pearson correlations between DYD and GEBV from three models for 152 Alpine and 100 Saanen goats**

**Trait**	**Alpine goats**		**Saanen goats**	
	**Multi-breed**	**Per breed**	**Multi-breed**	**Within-breed**
Milk yield	0.35	0.36	0.50	0.49
Fat yield	0.35	0.39	0.47	0.53
Protein yield	0.34	0.26	0.34	0.37
Fat content	0.47	0.55	0.53	0.65
Protein content	0.62	0.62	0.72	0.73
Somatic cell score	0.45	0.45	0.43	0.43
Udder floor position	0.52	0.53	0.65	0.65
Udder shape	0.51	0.51	0.51	0.50
Rear udder attachment	0.51	0.51	0.69	0.69
Fore udder	0.33	0.34	0.55	0.56
Teat angle	0.46	0.45	0.42	0.44

**Table 6:** **Breed-specific regression coefficients of DYD on GEBV from three models for 152 Alpine and 100 Saanen goats**

**Trait**	**Alpine goats**				**Saanen goats**			
	**Multi-breed**		**Per breed**		**Multi-breed**		**Within-breed**	
	**reg**	**SE**	**reg**	**SE**	**reg**	**SE**	**reg**	**SE**
Milk yield	0.54	0.12	0.65	0.14	0.84	0.15	0.89	0.16
Fat yield	0.49	0.10	0.72	0.12	0.67	0.13	0.93	0.14
Protein yield	0.52	0.12	0.45	0.12	0.54	0.15	0.56	0.15
Fat content	0.84	0.13	0.76	0.13	0.72	0.12	1.11	0.13
Protein content	0.98	0.10	1.02	0.10	1.19	0.12	1.27	0.12
Somatic cell score	0.68	0.11	0.72	0.12	0.66	0.14	0.69	0.14
Udder floor position	0.84	0.11	0.89	0.12	1.01	0.12	0.92	0.11
Udder shape	0.97	0.13	0.98	0.13	0.93	0.16	0.99	0.17
Rear udder attachment	1.15	0.16	1.21	0.13	1.70	0.18	1.71	0.18
Fore udder	0.54	0.17	0.62	0.17	1.05	0.13	1.11	0.14
Teat angle	0.99	0.18	0.99	0.19	0.85	0.16	0.93	0.16

The validation correlations estimated in this study were higher for the Saanen breed than for the Alpine breed for almost all traits, from 18% for fat and protein content to 65% for fore udder (Table 5). Similar results for the two breeds were obtained only for SCS, udder shape and teat angle. The absence of differences in phenotypic variances and DYD accuracies between the two breeds did not help to explain the difference in accuracies. However, this could be due to a higher inbreeding level in the Saanen breed (2.3% in Saanen vs 1.8% in Alpine) and a higher kinship coefficient between the training and testing sets (2.4% in Saanen vs 1.1% in Alpine, using genomic data). Thus, the larger training set size available for the Alpine breed did not counterbalance the smaller relationships between training and testing sets it had compared to the Saanen breed.

Table 6 reports regression coefficients of DYD on GEBV for each breed with the multi-breed and the within-breed models. Slopes obtained with the multiple-trait model were similar to those obtained with the multi-breed model (results not shown). As mentioned previously, almost all slopes of DYD on GEBV were less than 1, which indicates over-dispersion of GEBV. Regression coefficients were closer to 1 in Saanen than in Alpine goats, except for protein content, SCS, rear udder attachment and teat angle. Since the validation correlations obtained for the Saanen breed were higher than for the Alpine breed, the best slopes were obtained for the Saanen breed. Differences between the two models were greater for regression coefficients than for accuracies and ranged from 1% for udder shape for the Alpine breed to 69% for fore udder for the Saanen breed. Nevertheless, these differences were small compared to the high standard errors of the slopes.

Average model accuracies of the 148 candidates analyzed separately for each breed (results not shown) were little affected by the model used and were close to the accuracies analyzed by pooling Alpine and Saanen breeds together. Average model accuracies ranged from 0.62 for SCS, rear udder attachment and fore udder to 0.74 for protein content. Results on model accuracies were higher than expected given the small population size used in this study (86 Alpine and 62 Saanen individuals), and were slightly higher (by +1% for protein content to +8% for udder depth) for the Saanen than for the Alpine goats. The better results obtained for the Saanen breed compared to the Alpine breed could be explained by a higher inbreeding level [3] and kinship coefficient in the Saanen breed.

Genetic selection in the French breeding programs for Alpine and Saanen breeds is achieved through within-breed selection. Therefore, to compare the three models proposed in this study, we need to focus on the within-breed comparisons but because of the small size of the reference population (less than 400 males in each breed), multi-breed genomic evaluation has to be considered.

## Conclusions

This study compared three models (multi-breed, within-breed and multiple-trait) using a single-step approach for genomic evaluation. Quality of the predictions was similar for the three models, except for the dispersion of the GEBV, which was better with the within-breed model. The single-step approach resulted in higher prediction accuracy and over-dispersion of GEBV compared to the two-step approach. Average model accuracy for the candidates using a single-step approach outperformed the accuracy derived from pedigree-based parent average information from official evaluations, except for udder shape and teat angle. The best accuracies were obtained with the multi-breed model. Considering the small size of the population used in the within-breed model, accuracies were not expected to be high. A major gene or causal mutation specific to each breed (*DGAT1* and casein variant) could explain the good results obtained for the within-breed model. Based on prediction quality, there was no difference between the three models compared in this study. The most convenient model for genomic evaluation in French dairy goats would be the multi-breed model using a single-step approach. This model is the easiest to implement since it requires just one evaluation instead of two (multi-breed *vs* within-breed) and less computing time than the multiple-trait model. However, the dispersion of the GEBV indicates that improvements are needed before this model can be viably implemented in official evaluations.
